# Depressed as Freshmen, Stressed as Seniors: The Relationship between Depression, Perceived Stress and Academic Results among Medical Students

**DOI:** 10.3390/bs8080070

**Published:** 2018-08-03

**Authors:** Magdalena Iorga, Corina Dondas, Cristina Zugun-Eloae

**Affiliations:** 1 Department of Behavioral Sciences, University of Medicine and Pharmacy Grigore T. Popa of Iași, Iasi 700115, Romania; 2 Department of Vocational Counselling, University of Medicine and Pharmacy Grigore T. Popa of Iași, Iasi 700115, Romania; corinadondas@gmail.com; 3 General Medicine Faculty, University of Medicine and Pharmacy Grigore T. Popa of Iași, Iasi 700115, Romania; cristina.zugun@yahoo.com

**Keywords:** medical student, depression, perceived stress, academic results

## Abstract

Research in the field has identified the presence of stress and depression among medical students. However, no other study has pointed out the differences between years of study. The objectives of the study are to identify the levels of stress and depression among medical students and to point out the relationship between these two variables. **Methods**: The cross-sectional study gathered data regarding socio-demographic characteristics, depression, self-identified psychological and physical symptoms during stressful periods and perceived stress among medical students in a university in Romania. Statistical analysis was performed using IBM SPSS Statistics v23. For comparative analysis the *t-test for independent samples* and *one-way ANOVA* was used and for correlational analysis, Pearson and Spearman correlations was used. **Results**: Freshmen are the most depressed and graduating students are the most stressed medical students. Statistical analysis reveals an average score of perceived stress (M = 17.31 ± 6.79) and mild-moderate depression (M = 10.11 ± 7.69). Women are more prone to symptoms of depression. Students enrolled in the third year of study are the least depressed and the least stressed. Perceived stress is significantly positive correlated with depression and negative in strong correlation with the number of course credits received. More than half of students experience an increased rate of anxiety and consumption of alcohol, coffee, sweets or cigarettes during stressful academic periods. Over 60% declare themselves satisfied with their academic results. **Conclusions**: Strategies to diminish the level of stress and depression among medical students are necessary. Psychological support and educational counseling should start from admission, since freshmen experience the highest rate of depression.

## 1. Introduction

As the lengthiest training period, education in general medicine requires a strong personality structure, the strength to do hard work and willingness to gain professional and communication skills to ensure one’s career success [1]. The challenge of the amount of information which must be assimilated, the seriousness of the practical (critical or life-and-death) situations which must be faced, as well as the long period of time required to be trained in the field make the life of a medical student very different from the period of training for other professions. One’s limitations and resources are often tested, and overcoming the former makes the future doctor a good professional.

Medical academic training is focused mainly on solving patients’ medical problems and offering support to them and their families, but few courses in the curricula teach students how to cope with burnout, how to tackle problems related to professional satisfaction or how to identify coping mechanisms which will help them overcome difficult moments [2,3,4].

Students more often experience depressive symptoms than the general population and higher rates were identified among medical students, compared to students from other specialties [5]. In a recent study by Dyrbye et al. [6] it was shown that medical students and residents were more likely to exhibit symptoms of depression than the control sample. Studies across several countries have demonstrated high rates of depression among medical students ranging from 15–24% in USA [7], 29.5% in Turkey [8], 30.6% in Cameroon [9], 37.2% in Malaysia [10], 51.3% in India [11]. Gender was found to be strongly related to the level of depression among female medical students and doctors, females having higher rates of depression compared to males [12,13]. Increased levels of stress are more common in medical students than in students from other academic specialties [14]. 

Stress and depression are closely related, and they have negative impacts on academic achievement and results and increase the rate of errors and determine psychological and physical symptoms and suicidal thoughts [14,15]. The main source of stress among medical students was found to be academic studies [16].

The aim of the present study is to identify the level of perceived stress and the rate of depression among medical students from all six years of study. Second, we wanted to find the relationship of these variables with socio-demographic characteristics, academic results and self-identified psychological and physical manifestations. Third, a comparative analysis considering gender and year of study could reveal important results useful to consider in the process of coping with stress and depression. 

This cross-sectional study is the first one in the field developed in Romania, presenting a comparative analysis of medical students from all years of study. No other research was found in the scientific literature to show the differences between students’ level of depression, perceived stress and academic satisfaction, comparing subjects from each year of study; most of the studies focused on a single year of study [8], few cross-sectional studies included too small a number of students or they did not present results that compared all years of study [15] so based on these facts, our study also has an exploratory nature.

## 2. Materials and Methods

### 2.1. Study Design and Recruitment of Participants

Ethical approval was granted by the Research Ethics Committee of the “Grigore T. Popa” University of Medicine and Pharmacy in Iași, Romania. Students were informed about the goal of the study and informed consent was obtained from all participants prior to the beginning of the study. The research was performed between July 2016 and February 2017. All participants are enrolled at the “Grigore T. Popa” University of Medicine and Pharmacy in Iași, Romania, and come from all 6 years of study. The subjects voluntarily filled in the socio-demographic and academic information data and they had to answer to the questionnaires. No inducements were given to participants. Excluding criteria: questionnaires filled in after February 2017 or incompletely filled in. The response rate was about 92.46% (601 out of 650).

### 2.2. Questionnaires

#### 2.2.1. Psychological Tools

Two assessment tools are available and were used to measure the level of perceived stress and depression among university students. For this research, to identify the level of perceived stress among the questioned medical students the *Perceived Stress Scale—10* (PSS-10) developed by Cohen & Williamson [17] was used. The items of this scale evaluate the degree to which people find that life is unpredictable, uncontrollable, or overloaded and the responses are recorded on a Likert scale from 0 (never) to 4 (very often). It has been used in a wide range of research with general and clinical populations and the results have indicated adequate reliability (α = 0.82, test-retest, r = 0.77), validity, and sensitivity of the 10-item short version of the PSS [18].

To measure the level of depression the *BECK Depression Inventory* (BDI) was used [19]. The instrument contains 21 items. Each item is evaluated on a 4-point Likert-type scale from 0 to 3, the subjects having to choose the answer that suits best. The total score (from 0 to 63) represents the sum of the subscores for each item. This depression inventory is one of the most used instruments for assessing depression and could be used consecutively for identifying its level. The 21 symptoms were chosen from common psychological and psychiatric symptomatology: mood, pessimism, sense of failure, lack of satisfaction, guilty feelings, sense of punishment, self-hate, self-accusations, self-punitive wishes, crying spells, irritability, social withdrawal, indecisiveness, body image, work inhibition, sleep disturbance, fatigability, loss of appetite, weight loss, somatic preoccupations and loss of libido. A meta-analysis of the BDI’s internal consistency performed on twenty-five years of use of this instrument [20] shows a mean coefficient alpha of 0.86 for psychiatric patients and 0.81 for non-psychiatric participants. The mean correlations of the BDI samples with clinical ratings for depression and the Hamilton Psychiatric Rating Scale for Depression (HRSD) were 0. 72 and 0.73, for psychiatric patients. For the non-psychiatric participants, the mean correlations of the BDI with clinical ratings for depression and the HRSD were 0.60 and 0.74. 

Cronbach’s alpha score in the present research for BDI inventory is 0.874 and 0.850 for PSS-10. The scores reveal a good internal consistency for both instruments used in this paper.

#### 2.2.2. Socio-Demographic and Academic Data

Due to the fact that this is the first cross-sectional study performed on depression and perceived stress among medical students in this university campus, several socio-demographic and academic data were gathered in order to explore and describe the sample: age, sex (male/female), year of study (from 1 to 6), environment (urban/rural), having a chronic disease (yes/no), family history of chronic disease (yeas/no), the number of siblings, accommodation (living with their parents, having their own apartment or living in a rented apartment). 

As for the self-reported academic data, the number of credits (points) obtained during the previous year of study (from a maximum of 600) and the number of failed exams were also considered. The number of credits and the number of failed exams were used as variables because they are an objective measure for academic performance. It is useful to evaluate the influence of depression and perceived stress on academic performance (which is different from academic satisfaction, measured by a different item).

The students were questioned if they had drop-out thoughts during their academic life. Also, they had to evaluate the level of satisfaction with their academic results on a 5-point Likert-type scale from 1 (*very unsatisfied*) to 5 (*very satisfied*).

#### 2.2.3. Self-Rated Physical and Psychological Symptoms

A pre-test was conducted among medical students to identify the most frequent psychological and physical symptoms experienced by them during stressful periods. The following variables were considered: abuse of substances like coffee/tea/sweets/cigarettes, changes in appetite, restlessness, agitated sleep, tremors, headache, anxiety, weight-related problems, insomnia, emotional exhaustion, nausea, arrhythmia, low self-esteem, physical exhaustion, hair loss, lumps in the throat, cold extremities, dermatological problems, diarrhea, gastric pain, abdominal pain, somnolence, fragile nails, frequent urination, back pain, onset of visual problems, motor tics, vomiting, nail biting, aggravating visual problems, fainting sensations, consumption of pills or drugs, irritable bowel syndrome and constipation. The list of self-evaluated physical and psychological symptoms was delivered to students and they had to check those who are usually experienced by them.

Students also had to mention some coping activities that they practice to diminish their level of stress during the periods of academic evaluations.

### 2.3. Statistical Analysis

The analysis of obtained data was processed with IBM SPSS Statistics v23. For descriptive analysis means and standard deviations were considered. For comparative analysis One-Way ANOVA, Mann-Whitney U Test and T-test for independent samples were performed, the statistical difference being defined as *p* < 0.05. Pearson and Spearman correlations were performed to find the relationship between variables. Regression analyses were performed to examine the relationship between academic performance and stress/depression symptom scores.

## 3. Results

### 3.1. Socio-Demographic Data

601 students (163 male and 438 female students) enrolled in all six years of study were included in the research. The fact that the number of female students included in the research is higher than that of male students is in accordance with the conclusions of gender statistics regarding medical students, which state that the number of male students in medical universities is higher than that of female students. This proportion is consistent with other medical universities and with the number of female doctors in the world [21]. The distribution of students accordingly to the year of study and sex is presented in Table 1.

Out of 601 students, 466 (77.54%) live in urban areas and 135 (22.46%) live in the countryside. 202 students are only children (33.6%) and almost half of students have one sibling (N = 291, 48.4%). The results relating to the other socio-demographic data are presented in Table 2.

### 3.2. Academic Data

Almost ¼ of students (N = 150, 24.8%) declared that they have thought about dropping out of medical school. The rate is important and ranged between 20% and 35%, depending on year of study: 22.6% of first-year students, 28.1% of second-year students, 18.8% of third-year students, 35% of fourth-year students, 24% of fifth-year students and 19.6% of sixth-year students. 

Academic data relating to the number of credits, failed exams or the most stressful period are presented in Table 3.

Students were asked about their satisfaction with their academic performance and the results obtained in the most recent exam session. They had to evaluate the level of satisfaction on a 5-point Likert-type scale. 31 students (5.2%) declared that they were very dissatisfied with their academic results, 53 subjects (8.8%) were dissatisfied, several 137 (22.8%) questioned students declared that they were neither satisfied nor dissatisfied, 258 (42.9%) considered that they were satisfied and 122 students (20.15) were very satisfied with their academic performance. In total, over 60% of students declare that they were satisfied with their results in the final exams.

### 3.3. Psychological Data

Cronbach’s alpha score for the BDI inventory is 0.874 and 0.850 for PSS-10. The scores reveal a good internal consistency for both instruments used for this research. 

At the level of perceived stress, the subjects obtained an M = 17.31 ± 6.79. The score reveals an average score of perceived stress among medical students from all 6 years of study (cut-off scores for average PSS-10 is from 10 to 20).

The depression score for BDI is M = 10.11 ± 7.69, meaning that students have a mild-moderate level of depression (the cut-off score is between 10 and 18). 

Regarding self-rated psychological and physical symptoms, the most frequently identified symptoms and behaviors are: abuse of substances like coffee/tea/sweets/cigarettes (58.4%), changes in appetite (52.45%), restlessness (51.4%), agitated sleep (51.2%), tremors (45.8%), headache (44.3%), anxiety (43.1%), weight-related problems (42.8%), insomnia (42.6%), emotional exhaustion (40.9%), nausea (40.8%), arrhythmia (40.1%), low self-esteem (37.3%), physical exhaustion (36.1%), hair loss (34.9%), lumps in the throat (31.3%), cold extremities (29.8%), dermatological problems (25.3%), diarrhea (23.6%), gastric pain (23.0%), abdominal pain (22.3%), somnolence (19.5%), fragile nails (18.5%), frequent urination (18%), back pain (17.0%), onset of visual problems—such as visual fatigue or difficulty of visual focusing (16.6%), motor tics (16.6%), vomiting (15.6%), nail biting (11.5%), aggravating visual problems (15.3%), fainting sensations (15.1%), consumption of pills or drugs (15.1%), irritable bowel syndrome (10.6%) and constipation (7.8%). 

Students were asked what kind of activities they developed during stressful periods to diminish their level of stress. Among pleasant activities they mentioned: listening to music (20%), sleeping (17.8%), spending time in nature (13.3%), physical activity (13.1%), talking (7.7%), watching movies (7%) and reading books (5.5%).

### 3.4. Sex

The comparative analysis using the *t*-test revealed significant differences between male and females (see Table 4) considering the following items of BDI: self-accusations: t (599) = −2.04, *p* = 0.04; crying spells: t (599) = −3.57, *p* = 0.00; work inhibition: t (599) = −3.56, *p* = 0.00; fatigability: t (599) = −3.56, *p* = 0.00; loss of appetite: t (599) = −2.58, *p* = 0.01; weight loss: t (599) = −1.943, *p* = 0.05; somatic preoccupations: t (599) = −2.64, *p* = 0.00; loss of libido: t (599) = −2.51, *p* = 0.01; and the total score for depression is: t (599) = −2.54, *p* = 0.01. All mentioned scores show that women are more prone to showing symptoms of depression, even in the total score. Also, age seems to decrease the risk for depression; younger female students showed higher level of depression (R = −0.112 **, *p* = 0.006).

No significant difference was found between male and female students on perceived stress scores. The results for both psychological instruments are presented in Table 4, considering sex.

Using the Mann-Whitney test for independent samples, no significant difference was identified between male and female students considering their thoughts of dropping out and satisfaction with academic results. Applying an independent sample *t*-test, it was found that gender produces no difference nor for the number of failed exams or the number of credits obtained during the previous semester. 

### 3.5. Year of Study

One-Way Anova showed that for the perceived stress level, it was found that there are significant differences (F (5,595) = 6.170, *p* = 0.000) between subjects from the third year of study and those from the first, fourth and sixth years, with former students having lower scores. 

For depression scores, significant differences were identified between the first, third, fourth and sixth year of study: F (5,595) = 4.419, *p* = 0.001). For depression, the multiple comparison analysis performed with Bonferroni as post-hoc test showed the following results: between the first and third year of study (Mdif = 4.08, *p* = 0.001), between first and fourth Mdif = 9.36, *p* = 0.002, for years first and sixth Mdif = 3.72, *p* = 0.006). As for years 2 and 5, we have not obtained any significant differences.

For stress, the results are the following: first and third Mdif = 2.99, *p* = 0.014, for third and fourth: Mdif = −3.21, *p* = 0.005, for third and sixth Mdif = −4.42, *p* = 0.000, for fifth and sixth Mdif = −3.60, *p* = 0.006. The means and standard deviations for the two instruments are presented in Table 5.

Comparative analysis showed that students from the first year of study have the highest level of depression and those from the last year of study have the highest score in perceived stress. Moreover, our results showed that students enrolled in the third year of study are the least depressed and have the lowest scores in perceived stress compared to students from the other years of study. 

#### Comparative Analysis between Preclinical (First and Second) and Clinical (Third, Fourth, Fifth and Sixth) Years of Study

No difference was identified in perceived stress among medical students considering the preclinical and clinical years of study or gender. However, a significant difference was identified in the depression score between students from preclinical years (11.75 ± 8.16) and clinical years (9.26 ± 7.31), the level of depression among students from the first two years being significantly lower compared to the other years of study t (599) = 3.794, *p* = 0.000.

### 3.6. Correlational Analysis between Depression and Perceived Stress

For correlational analysis, the normality of data distribution was tested by using the Kolmogorov-Smirnov test. Spearman correlations were used because none of the considered variables had a normal distribution. The results revealed a significant positive correlation between PSS-10 and the total score of depression. The results are presented in Table 6*.*

### 3.7. Stress and Depression Scores and Academic Performance

Correlational analysis pointed out that there are significant negative correlations between number of course credits received and the total score for depression (R = −0.121 **, *p* = 0.004).

Satisfaction with academic results was identified being strongly correlated negatively with perceived stress (R = −0.152 **, *p* = 0.000) and depression (R = −0.243 **, *p* = 0.000). Also, a strong negative correlation was identified between the number of course credits received and PSS-10, meaning that higher level of perceived stress of medical students is correlated with lower academic results (see Table 6). A Bonferonni Correction was needed to verify that this result is statistically significant. The Bonferonni corrected *p* value is 0.002, meaning that the result is statistically significant.

Regarding drop-out thoughts, using Spearman correlations, the results shows a significant negative correlation with perceived stress ((R = −0.179 **, *p* = 0.000) and depression (R = −0.270 **, *p* = 0.000).

### 3.8. Regression Analysis

Linear regression is used for the regression analysis. The results are presented in Table 7*.*

For the first regression analysis we used the number of points in the last year as a predictor and the total score for PSS-10 and the total score for BDI as the dependent variables. The results show that the predictor explains 1.5% of the variance of the total score of PSS-10 and 1.3% of the total score for BDI. Even though these models are statistically significant (*p* < 0.005), the effects we obtained are a bit weak. The predictor number of points in the previous year has a negative effect on the dependent variable the total score for PSS-10 (b = −0.015; β = −0.123) and a negative effect on the dependent variable the total score for BDI (b = −0.016; β = −0.114). This could suggest that a higher number of points the students obtained in the previous year could predict a lower amount of self-reported stress and depression among the students. 

For the second regression the number of failed exams in recent years was used as a predictor and the total score for PSS-10 and the total score for BDI the dependent variables. The results show that the predictor explains 1.6% of the variance of the total score of PSS-10 and 5.6% of the total score for BDI. Also, in this case the effect of the predictor is weak, but it is statistically significant (*p* < 0.005). The predictor number of failed exams in recent years has a positive effect on the dependent variable the total score for PSS-10 (b = 0.989; β = 0.125) and a positive effect on the dependent variable the total score for BDI (b = 2.135; β = 0.239). The results suggest that even though the effect is small that the more exams the students have failed in the previous year, the higher the level of self-reported stress and depression would be for them.

## 4. Discussion

The results obtained by this research are very important. Firstly, as this is cross-sectional research, the differences between students across all six years of study are important for identifying the level of perceived stress and depression. The results obtained show that students in preclinical years of study have significantly higher scores in perceived stress and in depression. Also, the more failed exams they will have and the less course credits they will obtain, the more depressed and stressed they might be. It was identified that there is a significant positive correlation between perceived stress and all BDI items, which is an important result of this research, and has indicated eight domains of depression (sense of failure, lack of satisfaction, self-hate, self-accusations, crying spells, fatigability, weight loss and somatic preoccupations) strongly negatively correlated with academic success: the more present the symptom is, the fewer credits the students obtain. 

The results are congruent with the scientific literature, where the variables have been emphasized only in comparative studies which have considered a smaller number of students or fewer years of study. Dahlin et al. [15], comparing students from three years of study, found that medical students obtained higher depression scores compared to the general population, and the comparative analysis revealed that female students were more depressive than male students. 

The present study has identified a mild level of depression, similar to the levels resulting from research conducted in other medical universities in the world [11]. The comparative analysis showed that female students obtain higher scores in total depression and in some associated domains. In the present study, females reported higher scores compared to their male counterparts in depression and some of its dimensions and the results are consistent with other studies. Recent research by Iqbal et al. [11], evaluating 335 medical students, showed that female gender, younger age and a lower year of study are variables related to higher rates of depression. Some other studies identified that female medical students are more stressed compared to males. Regarding the level of stress, we found no difference between men and women.

The present research presents the dynamic of depression and perceived stress among medical students identifying different level for each year of study. These variables are deeply involved in students’ motivation for academic achievement [22]. The results showed that depressive symptoms and perceived stress related with the number of credits or failed exams. 

The lowest rate of dropping-out thoughts is identified among students in the third year of study, our suggestion being that they successfully graduate the first two years of theoretical study and hard work and, starting from the third year, they have more practical stages and they start studying specialties more than basic medicine courses. The higher rate of dropping-out thoughts obtained among students in the fourth year could be explained by the fact that, compared to other university studies, medical education takes longer, so former colleagues from high school or even friends have already graduated after 3 years (but medical students continue to be enrolled in school).

Socio-demographic and medical data seemed to have no influence on the level of depression or perceived stress. Some studies identified that home-related issues or family-related aspects have a small influence on the considered variables. For example, 6% of stress is related to family-related problems, compared to 60% of stressors related to academic courses [16].

The present study identified that younger female students enrolled in preclinical years are more suitable to show high level of depression. 

### Strengths and Limitations of the Study

The strengths of the study are represented by the large number of students enrolled in all six years of study and the response rate is high (over 90%). Therefore, the results obtained could be generalized for Romanian medical students, especially for those studying at our university, UMF “Grigore T. Popa”. The results bring new value to the other cross-sectional studies regarding depression and perceived stress among medical students, providing a comparative study considering the preclinical and clinical years [23]. 

One limitation of the study is the inclusion of only native Romanian students enrolled at a state university. International students were not targeted by the present research, so other factors such as ethnicity, acculturative stress or race that seem to be significant were not included [24,25]. A future study should focus on identifying different factors (cultural, religious, etc.) influencing the level of perceived stress and depression among this category of students. Also, because this research focused on a public medical university, some factors influencing the considered variables could be related to the national curricula for medical universities and could be different from other medical universities in the world. 

The second limitation is caused by the unequal distribution of subjects by sex. However, statistics data for 2017 also reported that female students are more numerous than male students in medical studies and this rate is kept for working physicians in many European countries [19]. Studies on medical students usually include more female students than males in their research [2,3,16,26,27,28].

The third limitation is related to the selection of subjects included in the research. The sample is not clinically diagnosed, and the relationship between stress and depressive symptoms may be different in the current sample from in a clinical sample.

The fourth limitation is caused by the type of the study. This is a cross-sectional study. Some arguments regarding causal relationships between stress and academic performance may be associated with precociousness. Longitudinal studies are expected to show more clearly the relationship between variables during academic years or after graduation. 

The results of the paper have a lot of implications both for medical education and human resource management in hospitals. Some studies in the field showed that managing self or self-discipline among other personality traits are important for coping with stress, dealing with difficult work tasks and increasing the level of job satisfaction [29,30,31]. Therefore, this information is important in vocational counselling sessions for both students and professionals working in the medical field [32,33,34]. 

## 5. Conclusions

The results of this research are important for both students and teachers. Medical students are exposed to an average level of perceived stress and mild-moderate depression. Depression and perceived stress are closely connected and their influence on academic results such as the number of failed exams and the number of credits received is indicated by this study. The highest rate of depression characterizes students from preclinical years (especially freshmen) and the highest rate of perceived stress is among students about to graduate. Students from the third year of medical studies seem to be the least stressed and depressed, whereas students from the fourth year are the most prone to thinking of dropping out. Age, gender and year of study are risk factors for depression. Mental health services, group support or psychological counseling should be provided especially to female students, also considering their year of study and their medical specialty choices. 

Academic staff should find ways to diminish the level of stress and depression among medical students and to provide psychological support and educational counseling for students to cope with these problems starting from admission, since our results suggest that the highest levels of depression occur among freshman students. 

## Figures and Tables

**Table 1:** The distribution of students accordingly to the year of study and sex.

Year of Study	Total	Male	Female	Age
	(N, %)	(N, %)	(N, %)	(M ± St. dev)
1	106 (17.64)	17 (2.83)	89 (14.81)	19.88 ± 1.12
2	96 (15.97)	30 (4.99)	66 (10.98)	21.13 ± 1.46
3	112 (18.64)	31 (5.16)	81 (13.48)	22.14 ± 0.84
4	110 (18.30)	26 (4.33)	84 (13.98)	23.03 ± 1.10
5	75 (12.48)	31 (5.16)	44 (7.32)	24.08 ± 0.73
6	102 (16.97)	28 (4.66)	74 (12.31)	25.11 ± 1.17

**Table 2:** Socio-demographic data.

Variables	N (%) or Mean (±SD)
sex	Male N = 163 (27.12)
	Female N = 438 (72.88)
age	22.49 ± 2.06
	(with a minimum of 19 and a maximum of 32)
number of children in the family	minimum of 1, maximum of 11
having chronic disease	61 (10.1%)
chronic disease among family members	158 (26.35)
Accommodation	on campus 156 (26%)
	at home 187 (31.1%)
	in rent apartments 187 (31.1%)
	in personal apartments 71 (11.85)

**Table 3:** Academic data.

transferable credits obtained in previous year (from 600)	513.80 ± 56.29
	(with a minimum of 60 and a maximum of 600)
most stressful time of the academic year	first second of finals N = 39 (6.5%),
	second session N = 57 (9.5%),
	both sessions of finals N = 460 (77.5%)
	summer stages N = 3 (0.5%)
number of failed exams during previous year	0.49 ± 86 (with a minim of 0 and a maximum of 5)

**Table 4:** Results for PSS-10 and BDI considering sex.

Instruments		Results	Male	Female
*BDI*	Mood	0.34 ± 0.58	0.36 ± 0.63	0.33 ± 0.56
	Pessimism	0.36 ± 0.53	0.31 ± 0.55	0.38 ± 0.52
	Sense of failure	0.34 ± 0.62	0.31 ± 0.63	0.34 ± 0.62
	Lack of satisfaction	0.58 ± 0.70	0.57 ± 0.66	0.59 ± 0.71
	Guilty feelings	0.42 ± 0.71	0.40 ± 0.68	0.43 ± 0.72
	Sense of punishment	0.35 ± 0.65	0.36 ± 0.57	0.35 ± 0.67
	Self-hate	0.32 ± 0.51	0.26 ± 0.48	0.35 ± 0.52
	**Self-accusations**	0.67 ± 0.69	0.57 ± 0.59	0.70 ± 0.72
	Self-punitive wishes	0.13 ± 0.40	0.14 ± 0.38	0.13 ± 0.41
	**Crying spells**	0.38 ± 0.79	0.21 ± 0.59	0.44 ± 0.85
	Irritability	0.60 ± 0.74	0.59 ± 0.74	0.60 ± 0.74
	Social withdrawal	0.49 ± 0.69	0.54 ± 0.70	0.47 ± 0.69
	Indecisiveness	0.63 ± 0.77	0.56 ± 0.74	0.66 ± 0.78
	Body image	0.38 ± 0.70	0.35 ± 0.72	0.39 ± 0.70
	**Work inhibition**	0.53 ± 0.66	0.39 ± 0.54	0.59 ± 0.70
	Sleep disturbance	0.68 ± 0.74	0.60 ± 0.78	0.71 ± 0.72
	**Fatigability**	0.77 ± 0.72	0.60 ± 0.68	0.83 ± 0.72
	**Loss of appetite**	0.51 ± 0.70	0.39 ± 0.64	0.55 ± 0.72
	**Weight loss**	0.64 ± 0.88	0.52 ± 0.85	0.68 ± 0.88
	**Somatic preoccupations**	0.62 ± 0.71	0.50 ± 0.70	0.67 ± 0.71
	**Loss of libido**	0.36 ± 0.68	0.26 ± 0.60	0.40 ± 0.71
	**Total score**	10.11 ± 7.69	8.79 ± 6.93	10.58 ± 7.91
*PSS-10*	Total score	17.31 ± 6.79	16.49 ± 6.38	17.61 ± 6.91

**Table 5:** Results for PSS-10 and BDI per year of study.

Year of Study	PSS-10	BDI
	M ± St. dev	M ± St. dev
1	18.00 ± 7.23	12.92 ± 0.73
2	17.11 ± 7.45	10.46 ± 0.77
3	15.00 ± 6.62	8.83 ± 0.71
4	18.22 ± 6.13	8.95 ± 0.72
5	15.82 ± 6.29	10.44 ± 0.87
6	19.43 ± 5.97	9.19 ± 0.75

**Table 6:** The results for the correlational analysis between PSS-10 and BDI total score.

BDI Total Score	Total Score PSS-10
Total score BDI	R = 0.532 **, *p* = 0.000
Number of transferable credits	R = −0.173 **, *p* = 0.000

** *p* < 0.001.

**Table 7:** Regression analysis using academic performance as predictors.

Dependent Variable		PSS-10	BDI
**Number of points obtained in the last year**	R^2^ adjusted	0.013	0.011
	ΔR^2^	0.015	0.013
	F (1,551)	8.469 **	7.206 **
**Number of failed exams in the last year**	R^2^ adjusted	0.014	0.056
	ΔR^2^	0.016	0.057
	F (1,593)	9.457 **	36.021 **

** *p* < 0.001.
